# Kinetic Analysis Suggests Evolution of Ribosome Specificity in Modern Elongation Factor-Tus from “Generalist” Ancestors

**DOI:** 10.1093/molbev/msab114

**Published:** 2021-04-19

**Authors:** Arindam De Tarafder, Narayan Prasad Parajuli, Soneya Majumdar, Betül Kaçar, Suparna Sanyal

**Affiliations:** 1 Department of Cell and Molecular Biology, Uppsala University, Uppsala, Sweden; 2 Department of Molecular and Cellular Biology, University of Arizona, Tucson, AZ, USA; 3 Lunar and Planetary Laboratory and Steward Observatory University of Arizona, Tucson, AZ, USA

**Keywords:** translation machinery, molecular evolution, EF-Tu, generalist, ancestral sequence reconstruction, fast kinetics, specificity

## Abstract

It has been hypothesized that early enzymes are more promiscuous than their extant orthologs. Whether or not this hypothesis applies to the translation machinery, the oldest molecular machine of life, is not known. Efficient protein synthesis relies on a cascade of specific interactions between the ribosome and the translation factors. Here, using elongation factor-Tu (EF-Tu) as a model system, we have explored the evolution of ribosome specificity in translation factors. Employing presteady state fast kinetics using quench flow, we have quantitatively characterized the specificity of two sequence-reconstructed 1.3- to 3.3-Gy-old ancestral EF-Tus toward two unrelated bacterial ribosomes, mesophilic *Escherichia coli* and thermophilic *Thermus thermophilus*. Although the modern EF-Tus show clear preference for their respective ribosomes, the ancestral EF-Tus show similar specificity for diverse ribosomes. In addition, despite increase in the catalytic activity with temperature, the ribosome specificity of the thermophilic EF-Tus remains virtually unchanged. Our kinetic analysis thus suggests that EF-Tu proteins likely evolved from the catalytically promiscuous, “generalist” ancestors. Furthermore, compatibility of diverse ribosomes with the modern and ancestral EF-Tus suggests that the ribosomal core probably evolved before the diversification of the EF-Tus. This study thus provides important insights regarding the evolution of modern translation machinery.

## Introduction

Protein synthesis is the fundamental step of gene expression. Genetic codes transcribed in the messenger RNAs form proteins in the ribosome with well-defined structural and functional properties. The protein synthesis efficiency governs the growth and survival of an organism in a particular environment (Dennis and Bremer 1974). Protein synthesis takes place at the translation machinery (referred as TM), one of the oldest molecular machines on Earth. It has been proposed that ribosome, the large macromolecular complex that lies at the epicenter of the extant translation machineries, emerged in the so-called RNA world (Fox 2010). It has been thought that a functional TM probably already existed in the last universal common ancestor, at least ~3.5 Gya (Fox 2010). The efficiency of protein synthesis relies on specific interaction between the ribosome and the translation factors. Yet, how the modern TM with all the associated translation factors, which interact specifically with the ribosome in a highly articulated fashion, has evolved across billions of years of life on Earth remains unclear.

**Fig. 1.**
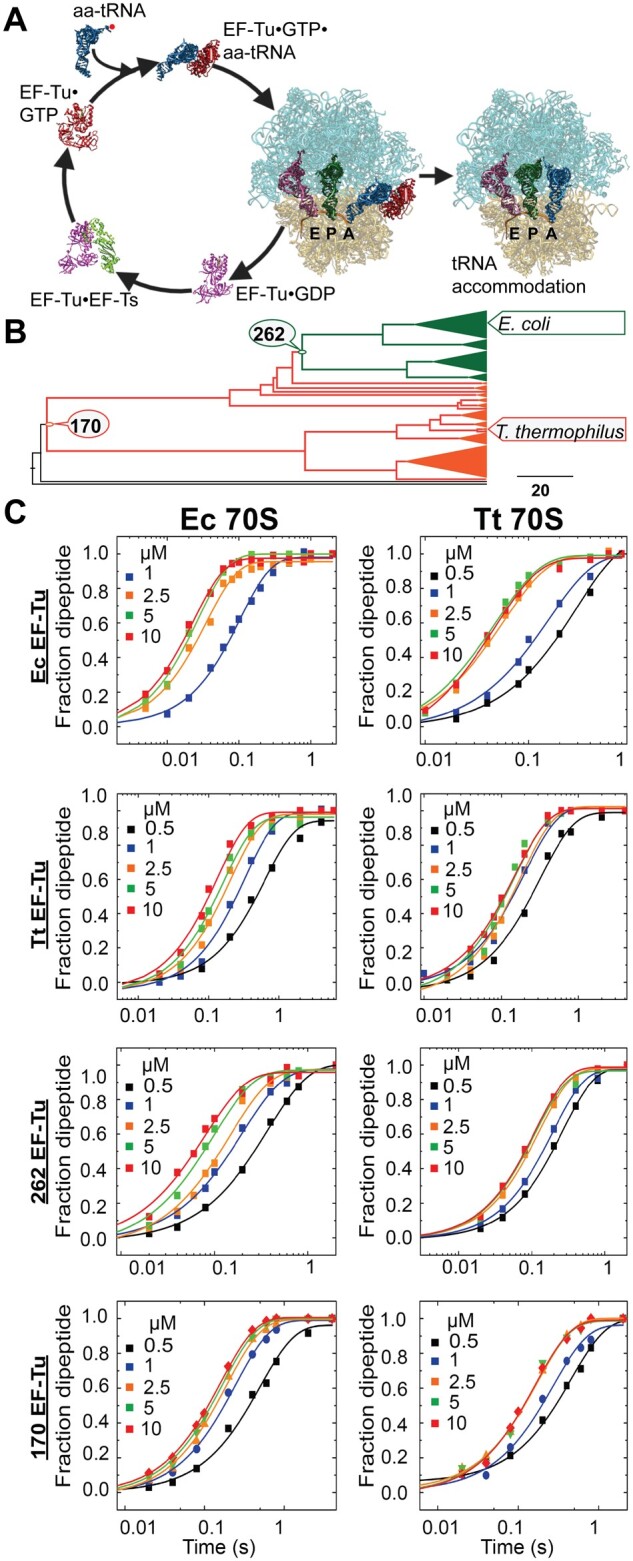
(*A*) The EF-Tu cycle in protein synthesis. EF-Tu•GTP binds to an aminoacyl tRNA (aa-tRNA) to form EF-Tu•GTP•aa-tRNA ternary complex, which binds to the A site of the ribosome. Upon codon-anticodon recognition EF-Tu hydrolyzes GTP and dissociates from the ribosome as EF-Tu•GDP after delivering the aa-tRNA. EF-Ts, which acts as a guanine nucleotide exchange factor for EF-Tu, binds to EF-Tu•GDP, and mediates exchange of GDP to GTP on EF-Tu, which reenters the cycle. PDBs 5WE4 (FislaGe et al. 2018), 6WD4 (Loveland et al. 2020), 6WDD (Loveland et al. 2020), 1EFC (Song et al. 1999), and 1EFU (Kawashima et al. 1996) were used in generating the figure. (*B*) EF-Tu phylogenetic tree indicating the nodes and taxa of the modern and ancestral protein homologs. The green circle represents the ancestral EF-Tu node 262 (262 EF-Tu). The orange circle represents the ancestral EF-Tu node 170 (170 EF-Tu). Although 262 EF-Tu is ancestral to alpha, beta, and gamma proteobacteria, including *Escherichia coli*, 170 EF-Tu is the most recent common ancestor to both *E. coli* and *Thermus thermophilus*. Descendant clades for the respective ancestral EF-Tus are highlighted in green and orange. The scale indicates amino acid replacements per site per unit evolutionary time. The tree was generated using sequences in reference (Gaucher et al. 2008; Kacar et al. 2017). (*C*) Kinetic characterization of the ancient and extant EF-Tus by dipeptide formation on 70S ribosomes; *Escherichia coli* (Ec 70S) and *Thermus thermophilus* (Tt 70S) at 37 °C. Time course of f[^3^H]Met-Leu dipeptide formation at varying EF-Tu concentrations (as indicated) on Ec 70S (left panel) and Tt 70S (right panel) (representative plots). The reactions were conducted in quench-flow instrument by rapid mixing of the 70S initiation complex with an elongation mix containing EF-Tu•GTP•aa-tRNA ternary complex in various concentrations (see Materials and Methods for details). The solid lines are single exponential fits of the experimental data.

The modern TM is a highly complex molecular system. Other than the RNA-based key components, namely the ribosome, tRNA, and mRNA, several nonribosomal translational protein factors play crucial roles in different steps of translation. Among these, elongation factor Tu (EF-Tu) is a vital housekeeping GTPase factor, which mediates the crucial step of delivery of the aminoacyl tRNAs (aa-tRNA) to the ribosome. Tallying up to 6% of the total protein in *Escherichia coli* (*E. coli*), EF-Tu is one of the most abundant proteins of the bacterial cells (Furano 1975). The prerequisite for elongation cycle is the formation of a stable ternary complex, where GTP bound EF-Tu binds to an aa-tRNA to form EF-Tu•GTP•aa-tRNA complex (Gordon 1969; Shorey et al. 1969; Miller and Weissbach 1977). EF-Tu brings the aa-tRNA to the A-site of the ribosome, where the triplet anticodon of the tRNA base-pairs with the codons of the mRNA. This cognate codon–anticodon interaction leads to GTPase activation in EF-Tu, which hydrolyzes GTP and dissociates from the ribosome in the GDP bound form (Loveland et al. 2017). In the cytoplasm, elongation factor-Ts (EF-Ts) acts as a guanine nucleotide exchange factor for EF-Tu, which catalyzes the exchange of GDP to GTP on EF-Tu, thereby allowing it to reparticipate in the elongation cycle (Lucas-Lenard and Lipmann 1971; Wieden et al. 2002) (fig. 1*A*). EF-Tu is a three-domain protein, where the G-domain is responsible for the GTPase activity and the domains II and III are involved in binding to the aminoacyl tRNAs. The GTPase and tRNA-recruitment functions of EF-Tu are highly ribosome dependent (Maracci et al. 2014), and hence, EF-Tu provides an excellent model system for studying the evolution of specificity of the translation factors to the ribosome.

It has been shown, that a strong selective constraint dictated by the host environment, controls the thermostability of EF-Tu from ancient times (Gaucher et al. 2003). Several ancestral EF-Tu variants, dating back to approximately 3.5 Gy, have been reconstructed using a methodology referred to as ancestral sequence reconstruction (Gaucher et al. 2003). Ancestral sequence reconstruction allows inferring ancestral sequences using phylogenetic reconstruction, the resurrected proteins are thereafter revived in the laboratory for structural, biophysical, and functional characterization (Zuckerkandl and Pauling 1965; Gaucher et al. 2003; Liberles 2007; Hochberg and Thornton 2017; Garcia and Kaçar 2019). The resurrected EF-Tus depicted a strong correlation between their thermostability and the proposed palaeotemperature trend of the ancient Earth’s environment, between approximately 3.5 and 0.5 Ga (Gaucher et al. 2008). These ancestral EF-Tus were later characterized in the reconstituted *E. coli* and *Thermus thermophilus* (*T. thermophilus*) translation systems. Using green fluorescent protein as a reporter it was demonstrated that the distant ancestors of proteobacterial EF-Tu were capable of synthesizing proteins in both the translation systems (Zhou et al. 2012). However, a recent study demonstrated that the *E. coli tuf* gene (encoding EF-Tu) has a limited functional interchangeability with its ancestral and modern homologs (Kacar et al. 2017). These findings thus open up an interesting possibility for studying the specificity of the ancestral and modern EF-Tus for the ribosomes with quantitative fast-kinetics experiments.

In this study, we explore for the first time, the evolution of ribosome specificity of the bacterial translation factors, with two ancestral EF-Tus as the model system. One is 262 EF-Tu (renamed as AnEF6 in Kacar et al. [2017]), which is an approximately 1.3-Gy-old nodal EF-Tu and the last common ancestor of the alpha-, beta-, and gammaproteobacteria (fig. 1*B*). The second one is 170 EF-Tu (renamed as AnEF3 in (Kacar et al. 2017), which is an approximately 3.3-Gy-old nodal EF-Tu and the most recent common ancestor to both *E. coli* and *T. thermophilus* classes (fig. 1*B*). Sequence alignment demonstrates that both the ancestral EF-Tus possess significant sequence similarity to the modern EF-Tus (supplementary fig. 1, Supplementary Material online). The nonconserved residues are distributed in all three domains of EF-Tu (supplementary video 1, Supplementary Material online). Using state-of-the-art quench flow-based dipeptide formation assay, we have characterized these two ancestral EF-Tus for their specificity towards the mesophilic *E. coli* ribosomes (Ec 70S) and the thermophilic *T. thermophilus* ribosomes (Tt 70S). Native EF-Tus from these two species were also tested for comparison. Furthermore, how the specificities of the thermophilic modern and ancient EF-Tus respond to the increase in temperature have also been monitored. Our study thus provides a comprehensive picture of evolution of specificity in EF-Tu for the ribosome, and reveals that the EF-Tu proteins likely evolved from a generalist, functionally promiscuous, ancient ancestor. Moreover, our results also hint toward the early evolution of the functional core of the ribosome.

## Results

### Ancestral EF-Tus Show Similar Specificity for Modern Mesophilic and Thermophilic Ribosomes

In enzyme kinetics, the “specificity constant” is denoted by the parameter *k*_cat_/*K*_M_, which reflects the preference of an enzyme for different substrates. *k*_cat_, the catalytic constant, reflects the maximum rate of the enzyme activity, whereas *K*_M_ is the Michaelis constant, which is the substrate concentration at the half-maximal catalytic rate. According to the Michaelis–Menten (M–M) model of enzyme kinetics, the higher the specificity constant, the more the preference for the substrate.

**Fig. 2.**
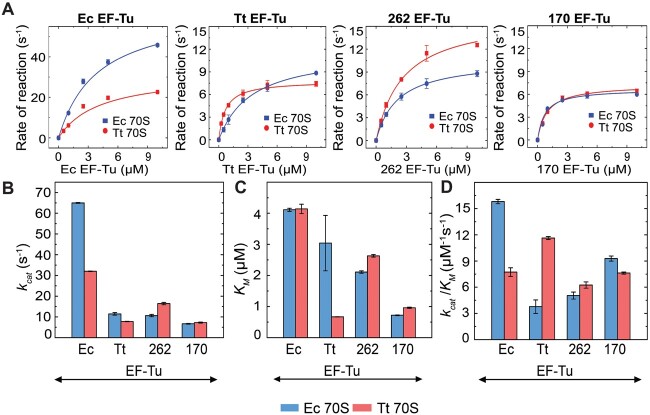
Michaelis–Menten parameters of the ancient and modern EF-Tu variants. Top row: Plots for the rates of fMet-Leu dipeptide formation (fig. 1*C*) against EF-Tu concentration with (*A*) Ec EF-Tu, (*B*) Tt EF-Tu, (*C*) 262 EF-Tu, and (*D*) 170 EF-Tu on 70S ribosomes; *Escherichia coli* (Ec 70S, blue) and *Thermus thermophilus* (Tt 70S, red). The Michaelis–Menten parameters are estimated by fitting the data with hyperbolic function using Michaelis–Menten equation. Bottom row: Comparison of the EF-Tu variants for (*B*) maximal rate (*k*_cat_), (*C*) Michaelis–Menten constant (*K*_M_), and (*D*) ribosome specificity (*k*_cat_*/K*_M_) on Ec 70S (blue) and Tt 70S (red), respectively. Error bars represent SEM.

By conducting presteady state kinetics of dipeptide formation, we have compared the ancestral EF-Tus 262 and 170, and the modern EF-Tus from *E. coli* (Ec EF-Tu) and *T. thermophilus* (Tt EF-Tu), for their specificity toward the Ec 70S and the Tt 70S ribosomes. For that, an initiating ribosome programmed with MLL mRNA and carrying f[^3^H]Met-tRNA^fMet^ in the P-site was rapidly mixed at 37 °C in a quench-flow apparatus with an elongation mixture containing the EF-Tu variants in ternary complex with Leu-tRNA^Leu^ and GTP. The f[^3^H]Met-Leu dipeptides formed over time are plotted and the curves are fitted with single exponential function (fig. 1*C*). Further, the rates obtained with different EF-Tu concentrations are fitted with a hyperbolic function (fig. 2*A*) to estimate the M–M parameters *k*_cat_, *K*_M_, and *k*_cat_/*K*_M_ (fig. 2*B*–*D* and table 1).

**Table 1.** Michaelis–Menten Parameters for f[^3^H]Met-Leu Dipeptide Formation by the EF-Tu Variants on Ec 70S and Tt 70S.

Ribosome	EF-Tu	Michaelis–Menten Parameters		
		*k*_cat_ (s^−1^)	*K*_M_ (μM)	*k*_cat_ */K*_M_ (s^−1^ μM^−1^)
Ec 70S	Ec EF-Tu	65.0 ± 0.18	4.1 ± 0.05	15.8 ± 0.25
37 °C	Tt EF-Tu	11.5 ± 0.61	3.0 ± 0.89	3.8 ± 0.77
	262 EF-Tu	10.6 ± 0.56	2.1 ± 0.04	5.0 ± 0.40
	170 EF-Tu	6.7 ± 0.15	0.7 ± 0.01	9.3 ± 0.29
Tt 70S	Ec EF-Tu	32.0 ± 0.1	4.1 ± 0.15	7.7 ± 0.49
37 °C	Tt EF-Tu	7.8 ± 0.07	0.7 ± 0.005	11.6 ± 0.17
	262 EF-Tu	16.4 ± 0.59	2.6 ± 0.04	6.3 ± 0.36
	170 EF-Tu	7.3 ± 0.26	1.0 ± 0.02	7.6 ± 0.12
Tt 70S	Tt EF-Tu	25.9 ± 0.277	2.5 ± 0.155	10.4 ± 0.666
50 °C	262 EF-Tu	22.4 ± 0.245	3.4 ± 0.115	6.5 ± 0.181
	170 EF-Tu	31.0 ± 0.6	2.7 ± 0.07	11.5 ± 0.93

Note.—The *k*_cat_, *K*_M_, and *k*_cat_/*K*_M_ parameters were estimated from hyperbolic fits of the observed rates of dipeptide formation on Ec 70S and Tt 70S. The data presented are average of at least three independent experiments with SEM. Due to the thermounstable nature, experiment with Ec EF-Tu was not conducted at 50 °C.

In order to ensure that the dipeptide experiments are not influenced by the affinity of the EF-Tu variants to the *E. coli* Leu-tRNA^Leu^ used here, we conducted nitrocellulose filter binding assay with [^3^H]Leu. All EF-Tu variants showed comparable counts retained on the filter. These results presented in supplementary figure 2, Supplementary Material online, thus confirm similar and saturated binding of Leu-tRNA^Leu^ to all EF-Tus under our experimental conditions. This result thus ensures that the kinetic parameters estimated in the dipeptide experiments are reflective of EF-Tu and ribosome specificity and are not influenced by the affinity of the EF-Tu variants to Leu-tRNA^Leu^.

As summarized in figure 2*B*–*D* and table 1, the *k*_cat_/*K*_M_ of dipeptide formation for Ec EF-Tu decreases from 15.8 μM^−1^s^−1^ on its native Ec 70S to 7.7 μM^−1^s^−1^ on Tt 70S. This 50% reduction in the ribosome specificity of Ec EF-Tu can be attributed to a decrease in *k*_cat_ from 65 s^−1^ on native Ec 70S to 32 s^−1^ on the nonnative Tt 70S ribosomes. Interestingly, the *K*_M_ values of Ec EF-Tu do not change for the two ribosomes. Similarly, the Tt EF-Tu also shows a higher *k*_cat_/*K*_M_ value of 11.6 μM^−1^s^−1^ on its native Tt 70S compared with 3.8 μM^−1^s^−1^ on Ec 70S. In this case, a reduced *K*_M_ of 0.7 μM on the Tt 70S compared with 3 μM on Ec 70S is a major contributor to the 3-fold decrease in its ribosome specificity. These results demonstrate a clear preference of the modern Ec and Tt EF-Tus for their respective ribosomes.

In stark contrast to the modern EF-Tus, 262 EF-Tu exhibits no preference for Ec 70S and Tt 70S as reflected by the comparable *k*_cat_/*K*_M_ values for both Ec 70S and Tt 70S (fig. 2 and table 1). Although 262 EF-Tu shows higher *k*_cat_ on Tt 70S (16.4 s^−1^), than on Ec 70S (10.6 s^−1^), the *K*_M_ for these two ribosomes also varies in a proportionate manner, resulting in similar *k*_cat_/*K*_M_ on both Ec 70S and Tt 70S (table 1). The 170 EF-Tu also presents similar *k*_cat_/*K*_M_ for Ec 70S and Tt 70S. Moreover, for this EF-Tu, both *k*_cat_ and *K*_M_ remain almost invariant irrespective of the Ec or Tt ribosomes (table 1). Thus, our quantitative estimates of specificity imply that the ancestral 262 and 170 EF-Tu do not have preference for the mesophilic or thermophilic bacterial ribosomes for dipeptide formation. Our results thus echo the notion that ancient enzymes can act on multiple substrates with similar efficiency, and that the substrate specificity of modern enzymes develops through billions of years of molecular evolution (Jensen 1976).

### The Specificity of the Thermophilic EF-Tu Variants Does Not Alter Significantly in the Temperature Range between 37 and 50 °C

**Fig. 3.**
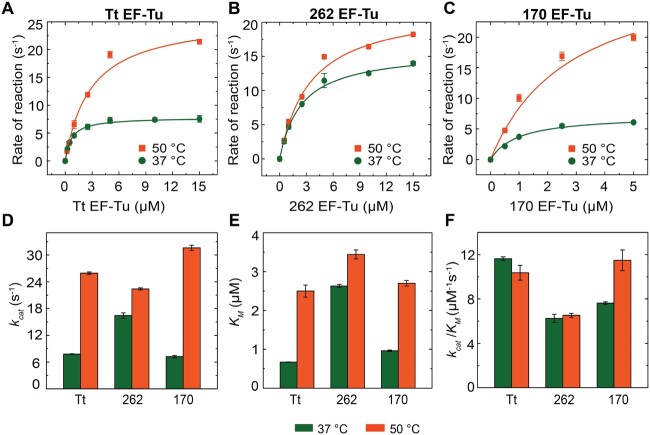
Kinetic efficiency of the thermophilic EF-Tus on Tt 70S at 50 and 37 °C. Top row: Plots for the rates of fMet-Leu dipeptide formation against EF-Tu concentration with (*A*) Tt EF-Tu, (*B*) 262 EF-Tu, and (*C*) 170 EF-Tu at 37 °C (green) and 50 °C (orange) on *Thermus thermophilus* (Tt 70S) ribosomes. The data are fitted with hyperbolic function using the Michaelis–Menten equation. Bottom row: Comparison of the thermophilic EF-Tu variants (Tt EF-Tu, 262 EF-Tu, and 170 EF-Tu) for (*A*) maximal rate (*k*_cat_), (*B*) Michaelis–Menten constant (*K*_M_), and (*C*) ribosome specificity (*k*_cat_*/K*_M_) at 37 °C (green) and 50 °C (orange), respectively. Error bars represent SEM.

In order to ascertain the effect of temperature on the M–M parameters of the thermophilic EF-Tu variants (Tt EF-Tu, 262 EF-Tu, and 170 EF-Tu), the dipeptide formation assay was conducted at 50 °C using Tt 70S (supplementary fig. 3, Supplementary Material online). The *k*_cat_, *K*_M_, and *k*_cat_*/K*_M_ parameters estimated from the M–M plots (fig. 3*A*–*C*) on Tt 70S were compared with those estimated at 37 °C, which are summarized in table 1 and figure 3*D*–*F*.

An increase in the rate of catalysis is observed for all the variants of EF-Tu as indicated by the increase in *k*_cat_, when compared with the same at 37 °C (fig. 3*D*). Modern Tt EF-Tu and ancient 170 EF-Tu show an appreciable increase in their *k*_cat_ from 7.8 to 25.9 s^−1^ and from 7.3 to 31.0 s^−1^, respectively. In comparison, the *k*_cat_ for ancient 262 EF-Tu shows only a small increase, that is, from 16.4 to 22.4 s^−1^. Interestingly, similar extent of increase is also observed in their respective *K*_M_ values (fig. 3*E*). At 50 °C, Tt EF-Tu and 170 EF-Tu show an increase in their *K*_M_ from 0.7 to 2.5 μM and from 1 to 2.7 μM, respectively. In contrast, the ancient 262 EF-Tu shows a marginal increase in its *K*_M_ from 2.6 to 3.4 μM. Consequently, the substrate specificity (*k*_cat_/*K*_M_) of the thermophilic EF-Tus show only negligible to small variation within the temperature range of 37 and 50 °C (fig. 3*F*).

### Dipeptide Formation Is a Read-Out for EF-Tu Activity on the Ribosome

Dipeptide formation involves two steps; EF-Tu-dependent delivery of the aminoacyl tRNA to the ribosome, which is followed by an EF-Tu independent step, peptide bond formation. GTP hydrolysis by EF-Tu separates these two steps as EF-Tu•GDP departs from the ribosome prior to peptide bond formation. In order to ascertain that the varied catalytic efficiencies in dipeptide formation by the tested EF-Tus originate from “EF-Tu-mediated” steps, and not from the “ribosome-mediated” peptide bond formation step, we have measured the rates of GTP hydrolysis and dipeptide formation in a single reaction starting from a ribosomal initiation complex. For that, preincubated EF-Tu in ternary complex with Leu-tRNA^Leu^ and [^3^H]GTP was mixed rapidly with preformed *E. coli* 70S initiation complex with f[^3^H]Met-tRNA^fMet^, in a quench-flow instrument. The reactions were quenched after desired times and the ribosomal complex was separated by centrifugation. The supernatant was analyzed for the proportion of [^3^H]GTP/GDP by separating on a Mono-Q column. The peptides were released from the ribosome pellet by KOH treatment, which were then analyzed in RP-HPLC.

**Fig. 4.**
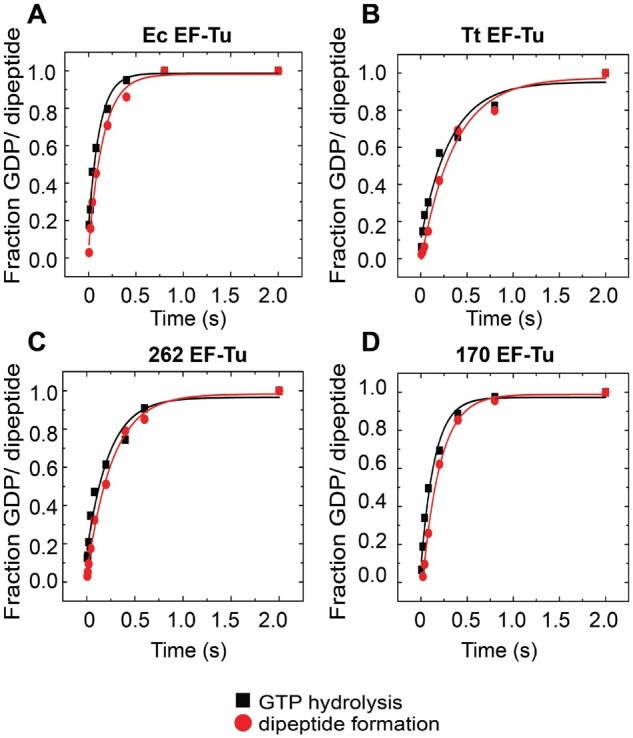
Mean time analysis for GTP hydrolysis on EF-Tu and dipeptide formation. The time course of EF-Tu-dependent GTP hydrolysis (black square) and fMet-Leu dipeptide formation (red circle) on Ec 70S followed in a single reaction with Ec EF-Tu (*A*), Tt EF-Tu (*B*), 262 EF-Tu (*C*), and 170 EF-Tu (*D*) measured in a quench-flow instrument. Solid lines represent single exponential fits to the data.

The amounts of GDP produced and dipeptide formed in the same reaction were plotted against time (fig. 4). The data points were fitted with single exponential function to estimate the rates. Further, the mean time of GTP hydrolysis (τ_GTP_) and the mean time of dipeptide formation (τ_dipep_) were estimated by reciprocal of the reaction rates. Finally, the mean time of peptide bond formation (τ_Pep_) was estimated by subtracting τ_dipep_ from τ_GTP_. As shown in figure 4, the dipeptide curves closely follow the GTP hydrolysis curves for all EF-Tu variants and produce similar values for τ_dipep_ and τ_GTP_ (table 2).

**Table 2.** Mean Time Analysis of GTP Hydrolysis and Dipeptide Formation with Different EF-Tu Variants on Ec 70S.

EF-Tu Variant	τ_dipep_ (ms)	τ_GTP_ (ms)	τ_pep_ (ms)
Ec EF-Tu	137.5 ± 7.5	104 ± 7	33.5 ± 0.5
Tt EF-Tu	384 ± 20	335.5 ± 12.5	48.5 ± 7.5
262 EF-Tu	248.5 ± 13.5	204 ± 22	44.5 ± 8.5
170 EF-Tu	200 ± 7	153.5 ± 5.5	46.5 ± 1.5

Note.—GTP hydrolysis and dipeptide formation were conducted in a single reaction with the EF-Tu variants on Ec 70S (fig. 4). The mean times, τ_dipep_, and τ_GTP_, are estimated from the reciprocal of the respective rates of the reactions. The mean time of peptide bond formation (τ_pep_) is estimated by subtraction of τ_dipep_ from τ_GTP_. The results in milliseconds (ms) are average values estimated from multiple independent experiments presented with SEM.

For all four EF-Tu variants tested here, the time course of dipeptide formation closely follows the time course of GTP hydrolysis (fig. 4) and produce very similar values for τ_dipep_ and τ_GTP_ (table 2). Among the four variants, on Ec 70S, the Ec EF-Tu shows the shortest τ_GTP_ of 104 ± 7 ms, whereas Tt EF-Tu displays the longest τ_GTP_ of 335.5 ± 12.5 ms. Ancestral EF-Tus, 262 EF-Tu, and 170 EF-Tu are found to be intermediates between the two modern EF-Tus, with τ_GTP_ 204 ± 22 ms and 153.5 ± 5.5 ms, respectively. Interestingly, τ_pep_, the difference between the mean times of dipeptide formation and EF-Tu mediated GTP hydrolysis, is about 40 ms irrespective of the τ_dipep_ and τ_GTP_ values for different EF-Tus (table 2). This is not unexpected as the peptide bond formation is not the function of the EF-Tus and rather an inherent function of the ribosome. Most importantly, τ_pep_ is significantly smaller than τ_GTP_, indicating that EF-Tu-mediated GTP hydrolysis is the rate-limiting step in the dipeptide reaction. The variation in the rates of the dipeptide formation with different EF-Tus is therefore reflective of their primary activity on the ribosome, that is, to escort the aminoacyl tRNAs to the ribosome and dissociate by GTP hydrolysis allowing tRNA accommodation and subsequent peptide bond formation. Thus, dipeptide formation is a valid read-out for the EF-Tu’s activity on the ribosome.

## Discussion

### Modern Translation Factors Evolved from Generalist Ancestors

How the TM evolved over time is a complex and yet unanswered question. Here, we studied the evolution of specificity in the TM by characterizing with fast-kinetics, two phylogenetically reconstructed ancient EF-Tus and compared those with modern bacterial EF-Tus from the thermophilic bacteria *T. thermophilus* and the mesophilic bacteria *E. coli*. Using dipeptide formation as a read-out, we have quantitatively determined specificity for those EF-Tu variants for bacterial ribosomes from the two above-mentioned thermophilic and mesophilic bacteria*.* Similar *k*_cat_/*K*_M_ values of the ancestral EF-Tus, 262 EF-Tu, and 170 EF-Tu for both modern Ec 70S and Tt 70S (figs. 1 and 2; table 1) demonstrate that they have similar specificities toward both mesophilic and thermophilic ribosomes, Ec 70S and Tt 70S, respectively. In contrast, the extant Ec and Tt EF-Tus show notably higher specificity to their native ribosomes. Through simultaneous monitoring of EF-Tu mediated GTP hydrolysis and dipeptide formation time course, we confirm that the variations in the catalytic rates in dipeptide formation originate solely from the EF-Tu mediated steps. Thus our results, for the first time, demonstrate with precise quantitative kinetics that the ancestral EF-Tus were “promiscuous” in regards to their compatibility to various ribosomes, whereas the modern EF-Tus are more “specific” toward their native ribosomes.

Our study reveals that Jensen’s “Generalist vs. Specialist” theory (Jensen 1976) applies to the evolution of specificity in TM. According to Jensen’s theory, ancestral enzymes were multifunctional generalists capable of withstanding larger variations of the substrates, whereas the modern ones are specialists in comparison (Jensen 1976). The “Generalist vs. Specialist” theory has so far been exemplified by various enzyme systems including serine proteases, steroid hormone receptors, mammalian serum paraoxonases and RuBisCo (Wouters et al. 2003; Eick et al. 2012; Bar-Rogovsky et al. 2013; Shih et al. 2016). Our results with ancient EF-Tu homologs indicate that a similar evolutionary mechanism may also apply to other components of the TM. Accordingly, the ancestral variants of the TM components in the ancient bacteria are likely to be generalists, compatible with diverse forms of their interaction partners that existed in the TM predecessor, the proto-TM, which could sustain protein synthesis under hypothetically limited resource and stringent conditions of the primitive Earth (Lunine 2006).

### Unchanged Ribosome Specificity of the EF-Tus in 37 and 50 °C

As shown in figure 4*F*, the specificity constant (*k*_cat_*/K*_M_) for 262-, 170-, and Tt EF-Tu remains unchanged when temperature is increased from 37 to 50 °C, although the catalytic constant (*k*_cat_) does. This is because the *K*_M_ values for these factors also increase proportionally. There are examples of several enzymes such as cellobiohydrolase, β-glucosidase, phosphatase, leucine-aminopeptidase, and tyrosine-aminopeptidase, whose catalytic efficiency increases with increasing temperature between 0 and 40 °C (Razavi et al. 2016). Alternatively, *k*_cat_*/K*_M_ values for cytidine deaminase and xylanase are shown to be insensitive to temperature variation in similar temperature range (Snider et al. 2000). It has been suggested that due to the compensatory effects of substrate binding and catalysis, the specificity constant did not change with temperature in these enzyme systems. Proportionate increase of *K*_M_ (substrate binding) and *k*_cat_ (catalysis) in the dipeptide experiments for the increase in reaction temperature from 37 to 50 °C suggests a similar explanation for unchanged ribosome specificity for the thermophilic EF-Tus. However, whether or not the balance between *K*_M_ and *k*_cat_, defining the ribosome specificity of the EF-Tus, retains in higher temperature range remains to be tested.

### Kinetic Characterization at 50 °C Indicates Lineage of the Ancestral EF-Tus

The dipeptide experiments performed at 50 °C demonstrate significant increase in catalytic activity (*k*_cat_) for Tt and 170 EF-Tu compared with 37 °C, whereas the 262 EF-Tu showed only a small increase in *k*_cat_ than in 37 °C (fig. 4*D* and table 1). These results suggest that 170 EF-Tu acts optimally at higher temperature similar to Tt EF-Tu, whereas 262 EF-Tu does not. Furthermore, the *K*_M_ for Tt and 170 EF-Tu are also similar and increase in a similar fashion for increase in the temperature from 37 to 50 °C (fig. 4*E* and table 1). Thus, 170 EF-Tu and Tt EF-Tu are more thermophilic in nature and functionally similar to each other, whereas 262 EF-Tu, is closer to the mesophilic Ec EF-Tu. This conclusion based on our kinetic data is also supported by the reported melting temperatures (*T*_m_) for these EF-Tu variants. The *T*_m_ for 170 EF-Tu is 66 °C, which is closer to the *T*_m_ 76 °C for the Tt EF-Tu, whereas the 262 EF-Tu has a lower *T*_m_ of 58 °C closer to the mesophilic EF-Tus (Gaucher et al. 2008; Zhou et al. 2012). Our kinetic data thus provide functional validation of the evolutionary relationship of the ancestral and the modern EF-Tus (fig. 1*B*).

### Correlation between Structural Flexibility and Catalytic Activity

The significantly low *K*_M_ values for the Tt EF-Tu and 170 EF-Tu in dipeptide formation at 37 °C (table 1) indicate that these EF-Tus bind very tightly to the ribosome at this temperature, which is much lower than their optimal temperature. It is known that the low-temperature adapted enzymes have significant conformational flexibility, especially in the region involved in catalysis (Závodszky et al. 1998; Akanuma et al. 2019). This flexibility is reduced in thermophilic enzymes and the reduced flexibility in the catalytic region entropically favors the enzymatic activity at high temperatures (Lam et al. 2011). In our study, upon increasing the temperature to 50 °C, *K*_M_ of Tt EF-Tu and 170 EF-Tu increase from 0.7 to 2.5 μM and from 1 to 2.7 μM, respectively. Hence, the low *K*_M_ values at 37 °C hint at the rigidity or lack of flexibility of the thermally adapted EF-Tus at lower temperatures. The lack of flexibility certainly favors tighter binding of these EF-Tus to the ribosome, but in turn limits their turnover capacity. This is reflected by their low *k*_cat_ values at 37 °C, which dramatically increase at 50 °C (fig. 4 and table 1). In contrast, a more mesophilic-like 262 EF-Tu shows *K*_M_ value comparable to Ec EF-Tu, which does not change much for increase of the temperature to 50 °C. Thus, our kinetic data suggest a correlation between the structural flexibility and the catalytic activity in EF-Tu, both optimize in an optimal temperature range. Below this range, a limited structural flexibility lowers catalytic activity of EF-Tu by tighter binding to the substrate, whereas increased flexibility above this range likely limits catalytic activity of EF-Tu by being error prone. Further increase in temperature leads to denaturation or aggregation of the EF-Tus causing loss of function. This evolutionary insight may assist future enzyme engineering and design studies.

### The Compatibility to Diverse EF-Tus Suggest an Early Evolution of the Ribosomal Core

Our data demonstrating that ancient EF-Tus exhibits functional promiscuity have implications for the origin and evolution of the ribosomal core. It is known that the ribosomal RNA (rRNA)-based peptidyl transferase center of the ribosome is highly conserved across the bacterial domain and even within minimal organelle ribosomes (Hsiao et al. 2009; Amunts et al. 2015). The peptidyl transferase activity of the ribosomes is retained after vigorous protein extraction treatments, but abolished when treated with ribonucleases (Noller et al. 1992). It has been proposed that translation initially evolved to extend the structural and functional capacities of an ancient ribozyme emerging from a precellular RNA World (White 1976; Noller 2004; Yarus 2011; Goldman and Kacar 2021). Our in vitro kinetic results show broad compatibility of the ribosomes toward diverse modern and ancestral EF-Tus in accordance with previous suggestions (Fahnestock and Rich 1971; Roesser et al. 1986; Sievers et al. 2004). Thus, our data suggest that the functional rRNA core of the ribosome likely evolved in primitive ancestors that preceded the diversification of EF-Tus and other translation factors.

In summary, our work provides evidence for a generalist ancestry of the bacterial TM, with promiscous translation factors in the ancient bacteria. The ribosome-specificity in the modern translation factors is probably the result of coevolution of both the ribosome and the factors. Future experiments will continue to shed light on this complex process that led to the evolution of the highly specific, modern molecular machines of protein synthesis.

## Materials and Methods

### Buffers and Translation Components

All experiments were conducted in HEPES-polymix buffer (pH 7.5) containing 5 mM HEPES (pH 7.5), 5 mM NH_4_Cl, 5 mM Mg(OAc)_2_, 100 mM KCl, 0.5 mM CaCl_2_, 8 mM putrescine, 1 mM spermidine, and 1 mM dithioerythritol (Koripella et al. 2015). Each reaction mixture contained 10 mM phosphoenolpyruvate, 0.05 μg ml^−1^ pyruvate kinase, and 0.002 μg ml^−1^ myokinase as energy pump components. The *E. coli* translation factors IF1, IF2, IF3, EF-Tu, EF-Ts, and leucine tRNA-synthetase (LeuRS) were overexpressed in *E. coli* BL21 (DE3) with C-terminal (His)_6_-tag and purified using Nickel-affinity chromatography (Histrap HP, GE Life Sciences).

The genes for Tt EF-Tu, 262 EF-Tu, and 170 EF-Tu were synthesized commercially and ligated into a pET21a vector between *Nde*I/*Xho*I sites, so that all these EF-Tus carry a C-terminal (His)_6_-tag. All EF-Tu variants were overexpressed in *E. coli* BL21 (DE3). For purification, respective lysates of the thermo-tolerant EF-Tus were incubated at 50 °C for 1 h and the cell debris together with denatured *E. coli* proteins were removed by centrifugation at 16,000 RPM for 1 h. Thereafter the proteins were purified using nickel-affinity chromatography. The protein concentrations were determined using Pierce 660 nm protein assay reagent.

*Escherichia coli* 70S ribosomes were prepared from JE28 strain as in (Ederth et al. 2009). The JE28 ribosomes carry (His)_6_-tags at the C-termini of the L7/12 proteins. *Thermus thermophilus* 70S ribosomes were prepared as described in (Selmer et al. 2006). f[^3^H]Met-tRNA^fMet^ and tRNA^Leu^ were prepared according to (Antoun et al. 2004).

XR7 mRNA with sequence AAGCTTGAAATTAATACGACTCACTATAGGGAATTCGGGCCCTTGTTAACAATTAAGGAGGTATTAA**ATGCTGCTGTAA**GAATTC encoding fMet-Leu-Leu-stop (MLL) (in bold) was prepared as in (Ge et al. 2018). [^3^H]GTP and [^3^H]Met were purchased from Perkin–Elmer. All other chemicals were purchased from either Merck or Sigma–Aldrich.

### Quench Flow-Based Dipeptide Formation Assay

An initiation mixture (IM) and an elongation mixture (EM) were prepared for each fMet-Leu dipeptide reaction. Leu was chosen as the second amino acid as tRNA^leu^ reading CUG codon is one of the most abundant tRNAs in bacteria. Moreover, the chosen second codon (CUG) has the highest frequency (∼50%) among all leucine codons in *E. coli* and other related bacteria. IM contained 70S ribosomes (0.5 μM *E. coli* or *T. thermophilus*), MLL mRNA (1 μM), f[^3^H]Met-tRNA^fMet^ (1 mM), GTP (1 mM), ATP (1 mM), and initiation factors IF1, IF2, and IF3 (1 μM each). In the EM, the final concentrations of EF-Tu and EF-Ts were varied from 0.5 to 10 μM keeping 1:1 ratio. The concentration of tRNA^Leu^ added in the EM was twice as much as the corresponding EF-Tu concentration. EM also contained GTP (1 mM), ATP (1 mM), leucine (200 μM), and LeuRS (0.5 μM). These two mixes were separately incubated for 25 min at 37 °C. Equal volumes of IM and EM were rapidly mixed in a temperature-controlled quench-flow instrument (RQF-3, KinTek Corp.). Here, the reaction was stopped at different predetermined time points by rapid addition of a quencher (17% final concentration of formic acid). The ribosomal complex was isolated with centrifugation at 14,000 RPM for 15 min at 4 °C. The peptides were then released by treating the ribosomal pellets with 0.5 M KOH. The ribosomes were pelleted again after adding 13 μl 100% formic acid. The released peptides in the supernatant were then analyzed using a RP-HPLC as in (Holm et al. 2016).

The rate of dipeptide formation was estimated by nonlinear curve fitting using a single exponential model using Origin 2018b software (OriginLab Corp.). The observed rates, *k*_dipep_, at different concentrations of EF-Tu, were plotted against EF-Tu concentration. The data points are fitted with hyperbolic function using the M–M equation to estimate *k*_cat_*, K*_M_, and *k*_cat_*/K*_M_ parameters. All experiments were done at least in triplicates and SEM was estimated using standard equation.

### Analyzing Mean Times of GTP Hydrolysis and Dipeptide Formation from a Single Reaction

Two reaction mixes IM and EM were prepared. IM contained 70S ribosomes (1 μM), MLL mRNA (2 μM), f[^3^H]Met-tRNA^fMet^ (2 mM), GTP (1 mM), ATP (1 mM), and initiation factors IF1, IF2, and IF3 (2 μM each). The EM contained EF-Tu (0.7 μM), tRNA^Leu^ (1.4 μM), [^3^H] GTP (1 mM), ATP (2 mM), leucine (200 μM), and LeuRS (0.5 μM). The mixes were rapidly mixed in quench flow and the reactions were quenched at definite time points with 17% (final) formic acid. The quench-flow products were centrifuged at 14,000 RPM for 15 min. The pellet fraction was processed for estimating dipeptide fraction as described above. The supernatant fractions containing [^3^H]GTP and [^3^H]GDP were analyzed using mono-Q column attached to HPLC (Holm et al. 2019). Both the data for dipeptide and GTP hydrolysis were plotted against time and fitted with single exponential functions using Origin 2018b software (OriginLab Corp.). The mean time for GTP hydrolysis (τ_GTP_) and dipeptide formation (τ_dipep_) were estimated from reciprocal of the respective rates. The mean time for peptide bond formation (τ_pep_) was estimated by subtraction of (τ_GTP_) from (τ_dipep_). All experiments were done at least three times. The error bars indicate SEM estimated using standard equation. 

## Supplementary Material

Supplementary data are available at *Molecular Biology and Evolution* online.

## Supplementary Material

msab114_Supplementary_Data
