# Supplementary material for: Kinetic Analysis Suggests Evolution of Ribosome Specificity in Modern Elongation Factor-Tus from “Generalist” Ancestors
Source: Mol Biol Evol. 2021 Apr 19;38(8):3436–44. doi: 10.1093/molbev/msab114 (PMC8321524; doi:10.1093/molbev/msab114)
Supplement: msab114_Supplementary_Data [file msab114_supplementary_data.zip › Supplementary_MBE revision 20210308.pdf]

Supplementary Information

Kinetic comparison of the ancestral and modern Elongation factor – Tus suggests generalist ancestry

Arindam De Tarafder[1], Narayan Prasad Parajuli[1], Soneya Majumdar[1], Betul Kacar[2,3], and Suparna Sanyal*[1]

[1] Department of Cell and Molecular Biology, Uppsala University, Box-596, BMC, SE-75124 Uppsala, Sweden

[2] Department of Molecular and Cellular Biology, University of Arizona, Tucson, AZ 85721

[3] Lunar and Planetary Laboratory and Steward Observatory University of Arizona, Tucson, AZ 85721

*To whom correspondence should be addressed: Email: suparna.sanyal@icm.uu.se; Tel: +46 18 471 4220

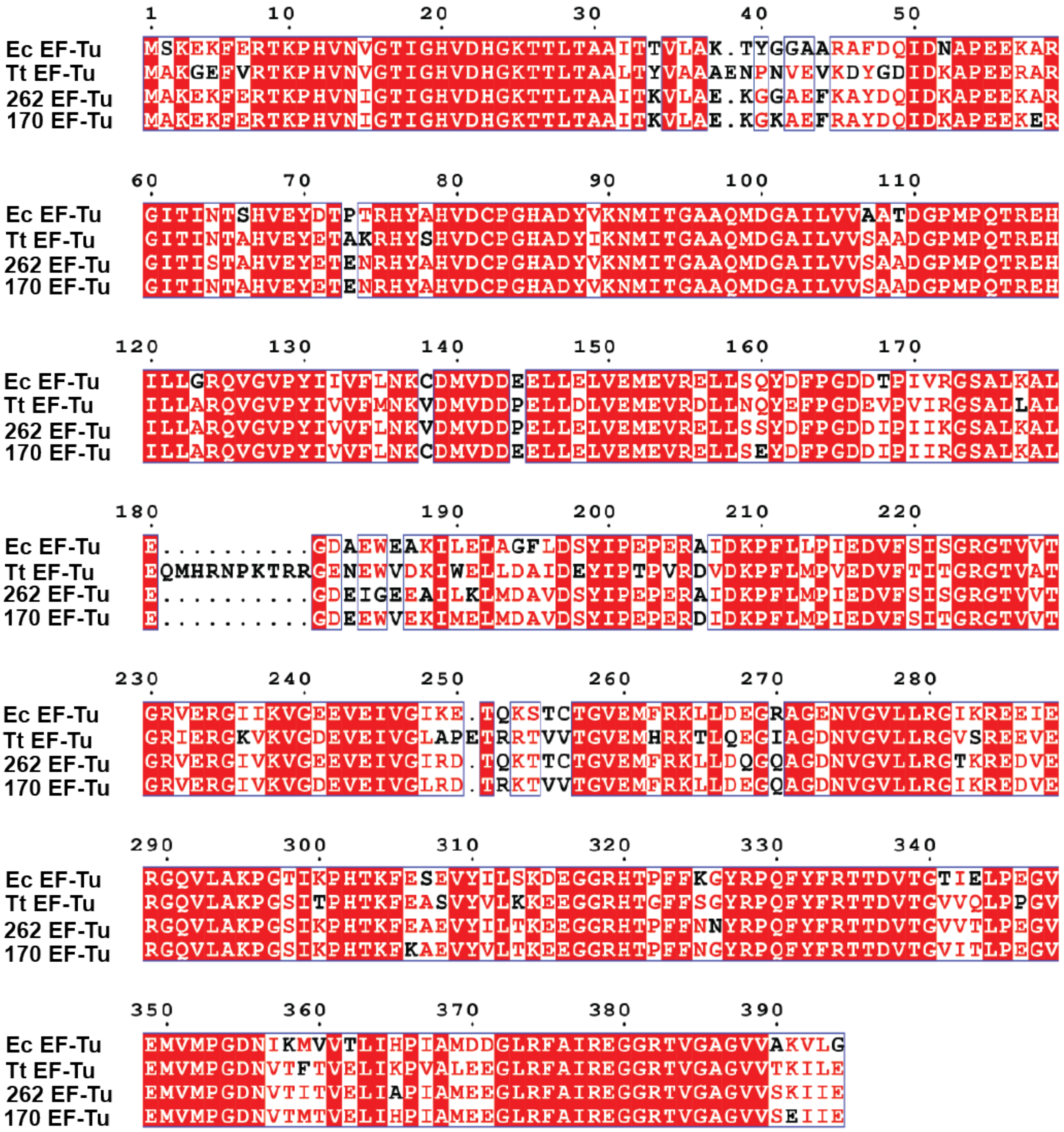

Supplementary Figure 1: **Sequence analysis of the EF-Tu variants**. Amino acid sequences for the ancient EF-Tus were obtained from ref.(Kacar et al. 2017) and the sequences for the modern Ec and Tt EF-Tus were obtained from the NCBI database. The sequences were aligned using Clustal Omega (Madeira et al. 2019) and the figure was generated in the ESPript 3.0 server (Robert and Gouet 2014). Strictly conserved amino acids are represented in white with red background. Chemically similar amino acids are represented in red with white background.

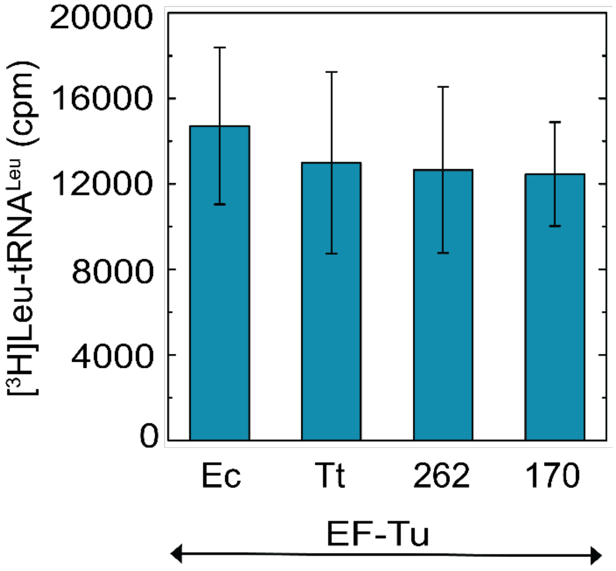

Supplementary Figure 2: Comparison of the relative binding of tRNA Leucine to the EF-Tu variants. Nitrocellulose filter binding experiments were carried out to compare the relative amounts of [^{3}H]Leu-tRNA^{Leu} bound to the EF-Tu•GTP•[^{3}H]Leu-tRNA^{Leu} ternary complexes formed at 37 °C with the four EF-Tu variants i.e. Ec EF-Tu(Ec) , Tt EF-Tu (Tt), 262 EF-Tu (262) and 170 EF-Tu (170).

Individual EF-Tu (2 µM) variant was mixed with tRNA^{Leu} (2 µM), EF-Ts (2 µM), GTP (1 mM), ATP (1 mM), [^{3}H] leucine (50 µM), LeuRS (0.5 µM) in HEPES-polymix buffer (pH 7.5) and was incubated for 25 min at 37 °C. Following incubation, the mixes were placed on ice. Thereafter they were filtered through nitrocellulose membranes presoaked in HEPES-ploymix buffer (pH 7.5) and washed to remove nonspecific binders. The radioactivity retained on the filter reflected the amount of EF-Tu•GTP•[^{3}H]Leu-tRNA^{Leu} formed, which was quantified using a Beckmann Coulter LS 6500 liquid scintillation counter and is represented as counts per minute (cpm). The background counts, obtained from the reactions without EF-Tu, were subtracted from individual datasets. All experiments were done in triplicates and the error bars represent SEM (Standard Error of Mean).

All EF-Tu variants showed similar counts confirming equivalent binding of [^{3}H]Leu-tRNA^{Leu} to those under our experimental condition. It also means that all dipeptide experiments conducted in this study with double amount of tRNA^{Leu} than EF-Tu is not influenced by the relative affinity of EF-Tu and tRNA^{Leu}.

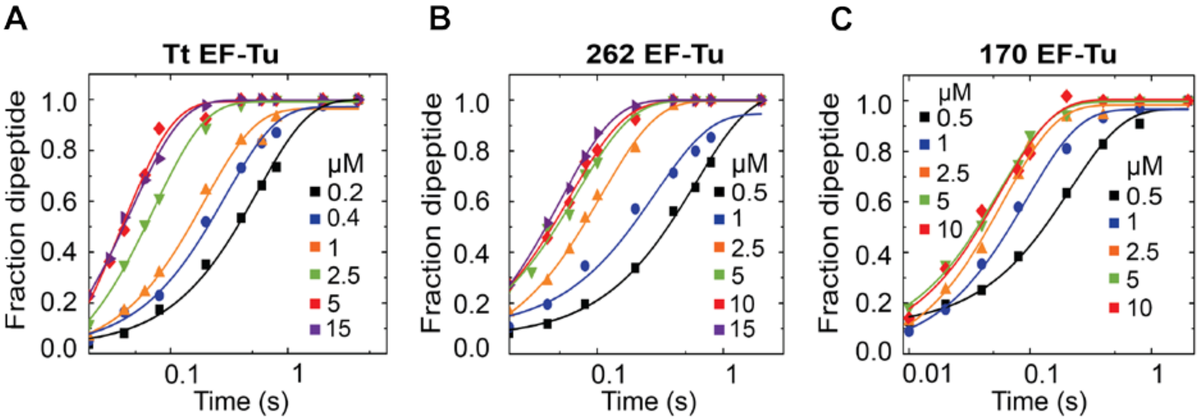

Supplementary Figure 3: **Kinetic characterization of the extant and ancient EF-Tu variants by dipeptide formation assay on Tt 70S at 50 °C.** Time course of f[^{3}H]Met-Leu dipeptide formation at varying concentrations of Tt EF-Tu (A), 262 EF-Tu (B) and 170 EF-Tu (C) on Tt 70S. The reaction was conducted in a quench flow instrument by rapid mixing of the 70S initiation complex with an elongation mix containing EF-Tu•GTP•aa-tRNA ternary complex in various concentrations (*see Materials and Methods* for details). The solid lines represent exponential fit to the data points. The plots are representative results from multiple independent experiments.

Supplementary Video 1

Structure of Ec EF-Tu (PDB ID : IDG1) depicting the distribution of different amino acids across the protein in comparison to the ancestral variants 262 and 170 EF-Tu. The regions with conserved or identical amino acids are in white, amino acids with chemically similar substitutions are in red, and different or non-conservative substitutions are in black.

References

Kacar B, Garmendia E, Tuncbag N, Andersson DI, Hughes D. 2017. Functional constraints on replacing an essential gene with its ancient and modern homologs. *mBio*. 8(4):e01276-17.

Madeira F, Park YM, Lee J, Buso N, Gur T, Madhusoodanan N, Basutkar P, Tivey ARN, Potter SC, Finn RD, et al. 2019. The EMBL-EBI search and sequence analysis tools APIs in 2019. *Nucleic Acids Res*. 47(W1):W636–W641.

Robert X, Gouet P. 2014. Deciphering key features in protein structures with the new ENDscript server. *Nucleic Acids Res*. 42:W320–W324.
